# SERF-MEG as a Functional Biomarker of Optic Nerve Injury: Agreement and Correlation with Visual Evoked Potentials

**DOI:** 10.3390/bioengineering13070830

**Published:** 2026-07-18

**Authors:** Helei Wang, Yuankun Qi, Yu Lou, Xu Zhang, Xinda Song

**Affiliations:** 1 School of Instrumentation and Optoelectronic Engineering, Beihang University, Beijing 100191, China; wanghelei326@buaa.edu.cn (H.W.); yuankunqi@buaa.edu.cn (Y.Q.); louyu@buaa.edu.cn (Y.L.); 2 Hangzhou National Institute of Extremely-Weak Magnetic Field Infrastructure, Hangzhou 310051, China; 3 State Key Laboratory of Traditional Chinese Medicine Syndrome, Hangzhou 310051, China

**Keywords:** SERF-MEG, optic nerve injury, visual evoked responses, electrophysiology, VEP

## Abstract

This study assessed the cross-modal agreement between spin-exchange relaxation-free magnetoencephalography (SERF-MEG) and conventional visual evoked potentials (VEPs) in patients with optic nerve injury using comparable pattern-reversal visual stimulation paradigms. Forty-five patients with optic neuritis (ON), ischemic optic neuropathy (ION), and traumatic optic neuropathy (TON) were enrolled, and a paired-eye design was applied to assess interocular electrophysiological changes between the affected and fellow eyes. Both modalities indicated consistent impairment of visual pathway function, reflected by reduced response amplitudes and prolonged latencies. For latency measures, MEG-derived M100 showed moderate directional concordance and a significant correlation with VEP P100, whereas amplitude measures demonstrated similar directional changes but limited quantitative correlations across modalities. Compared with the MaxP2P method, the channel-averaged method displayed higher stability and cross-modal concordance. These findings show that SERF-MEG can capture clinically relevant visual pathway impairment and may complement conventional VEP by providing additional information on cortical response patterns. Overall, SERF-MEG shows potential as an objective functional biomarker for neuro-ophthalmic assessment of optic nerve injury.

## 1. Introduction

Optic nerve disorders, including ON, ION, and TON, are major causes of visual dysfunction in neuro-ophthalmology. These disorders can damage retinal ganglion cell axons and disrupt the transmission of visual information along the visual pathway, from the optic nerve through the lateral geniculate nucleus to the occipital visual cortex, resulting in varying degrees of visual impairment [1,2,3]. Objective and reliable assessment of visual pathway function is therefore important for disease diagnosis, disease monitoring, and prognostic evaluation [4,5].

VEP is one of the most widely used electrophysiological tools for evaluating visual pathway function in clinical practice. Pattern-reversal VEP provides an objective measure of cortical responses to visual stimulation, and the P100 component is widely used because of its good reproducibility and relatively stable latency. P100 latency primarily reflects visual conduction time along the visual pathway, whereas its amplitude reflects the strength of synchronized cortical neuronal activity [6,7]. In various optic nerve disorders, prolonged P100 latency and reduced amplitude are established electrophysiological markers of impaired visual conduction and have been widely used to assess visual function in patients with ON, multiple sclerosis, and other optic neuropathies [8].

Despite its clinical utility, VEP has several inherent limitations. Because scalp electrodes record electrical potentials after volume conduction through the skull and surrounding tissues, VEP amplitudes are susceptible to variations in scalp impedance, electrode placement, and individual anatomical differences, resulting in substantial inter-individual variability [9]. Furthermore, the spatial resolution of conventional VEP recordings is limited, making it difficult to precisely localize the cortical generators of visual responses. Therefore, the development of more stable and objective functional assessment methods may further improve the evaluation of visual pathway function.

Magnetoencephalography (MEG) is a noninvasive functional neuroimaging technique that directly records the weak magnetic fields generated by synchronized neuronal activity. Compared with electroencephalography, MEG signals are minimally affected by the conductive properties of the scalp and skull, while providing millisecond temporal resolution and improved spatial localization [10,11]. During visual stimulation, MEG can record characteristic visual evoked fields (VEFs), enabling precise characterization of the temporal dynamics of cortical visual processing [12,13]. Among these responses, the M100 component, which predominantly originates from the primary visual cortex near the calcarine sulcus, has attracted considerable interest because its latency and amplitude are closely associated with visual input quality, conduction efficiency along the visual pathway, and the integrity of white matter pathways [14,15,16,17]. A functional biomarker is an objectively measured indicator that reflects a physiological function or a pathological functional change [18]. In this context, M100 latency may reflect visual conduction efficiency, whereas M100 amplitude may reflect the strength of the cortical visual response. These properties suggest that M100 may serve as a potential functional biomarker of visual pathway integrity.

In recent years, MEG has been increasingly applied in visual neuroscience research and has contributed substantially to the understanding of the dynamic cortical processing of visual information. Previous studies have used MEG to characterize early visual cortical responses and to examine how visual evoked fields vary with stimulus properties and visual input quality [19,20,21,22]. Hill et al. used OPM-MEG in two contrasting magnetic environments and obtained reproducible responses during visual–motor and face-processing tasks. Their findings showed that OPM-MEG could produce comparable temporal response patterns and source localization results across different recording sites [12]. These studies demonstrate the value of MEG for examining the timing and spatial distribution of cortical visual responses and support its potential use in the assessment of visual system disorders.

Compared with conventional MEG systems based on superconducting quantum interference devices (SQUIDs), non-cryogenic OPM/SERF-MEG systems do not require cryogenic cooling and may permit a shorter sensor-to-scalp distance, depending on the specific sensor and helmet configuration [23,24,25,26]. A shorter sensor-to-source distance may increase the measured neural signal amplitude and improve spatial sampling, although system-level performance also depends on sensor sensitivity, channel coverage, magnetic shielding, environmental noise, and data-processing methods. The absence of cryogenic cooling may reduce some infrastructure and maintenance requirements, but the overall cost and operational complexity depend on the complete system configuration. These characteristics support the potential use of SERF-MEG in neuro-ophthalmic research and functional assessment.

Despite the acknowledged advantages of MEG for assessing the spatiotemporal dynamics of cortical activity, its clinical utility in optic nerve disorders has not been fully established. In particular, it is unclear whether visual evoked responses recorded by SERF-MEG demonstrate sufficient directional concordance and correlation with conventional VEP measures to serve as valid indicators of visual pathway function. Furthermore, the cross-modal relationships between MEG-derived and VEP-derived measurements have not been comprehensively examined.

Therefore, the present study aimed to characterize visual evoked field abnormalities recorded by SERF-MEG in patients with optic nerve injury and to evaluate their directional concordance and correlation with conventional VEP measurements. Using a paired-eye design comparing affected and fellow eyes, we investigated the agreement in latency- and amplitude-related alterations revealed by the two electrophysiological modalities and further examined the cross-modal relationships between MEG-derived and VEP-derived metrics. Through these analyses, we aimed to evaluate the reliability of SERF-MEG as an objective functional assessment tool for optic nerve function and to assess its potential clinical utility in neuro-ophthalmology.

## 2. Methods

### 2.1. Research Design and Ethical Authorization

**Figure 1:**
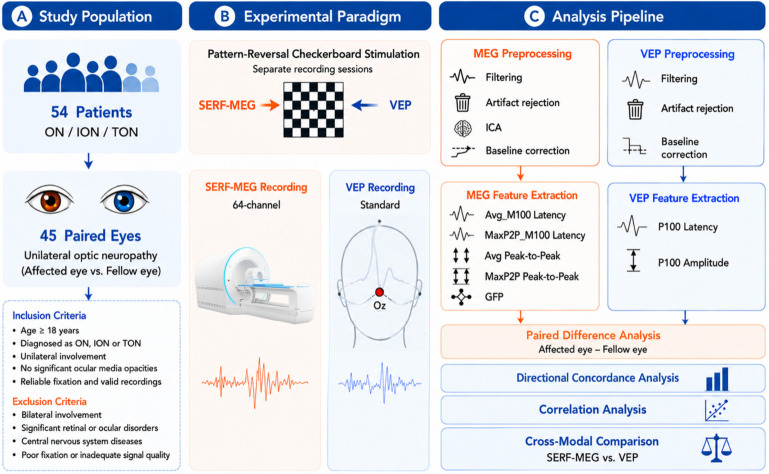
Schematic overview of the study workflow. The pipeline consists of three core components: (**A**) Study Population: 54 patients were enrolled, with 45 eyes meeting criteria for paired analysis (affected vs. fellow eye). (**B**) Experimental paradigm: SERF-MEG and conventional VEP recordings were acquired in separate sessions using comparable pattern-reversal checkerboard stimulation paradigms. (**C**) Analysis Pipeline: After preprocessing, key features were extracted: SERF-MEG yielded M100 latency, peak-to-peak amplitude, and GFP; VEP yielded P100 latency and amplitude. Subsequent analyses focused on paired-eye differences, directional concordance, correlation, and cross-modal comparison between SERF-MEG and VEP. Abbreviations: SERF-MEG, spin-exchange relaxation-free magnetoencephalography; VEP, visual evoked potential; GFP, global field power.

This study was designed as a prospective observational study aimed at characterizing changes in visually evoked magnetoencephalographic signals in patients with optic nerve injury and investigating the agreement and correlation between MEG-derived and VEP-derived indices. The overall study workflow is shown in Figure 1.

Patients were recruited from the Department of Ophthalmology at Qilu Hospital of Shandong University and included those diagnosed with optic nerve injury, including ON, ION, and TON. All diagnoses were established by experienced neuro-ophthalmologists according to relevant clinical criteria.

Exclusion criteria were as follows: (1) presence of significant corneal, lens, or retinal disorders affecting visual input; (2) concomitant central nervous system diseases; (3) inability to maintain stable fixation or cooperate with examinations; and (4) presence of excessive motion artifacts or suboptimal signal quality.

Given the substantial inter-individual variability in VEP amplitudes, a paired-eye design was adopted in this study. In patients with unilateral involvement, the affected eye was defined as the study eye, while the fellow eye served as an internal control. This within-subject design was used to minimize the influence of inter-individual differences in anatomical structure, scalp impedance, and overall signal amplitude. Patients with bilateral involvement or without clear unilateral abnormalities were excluded from the paired-eye analysis.

This study was approved by the Ethics Committee of Qilu Hospital of Shandong University (approval No. KYLL-202310-017-1) and was conducted in accordance with the Declaration of Helsinki. Written informed consent was obtained from all participants.

### 2.2. MEG and VEP Recording

MEG signals were acquired using a SERF-MEG system (LMEG-64A, ZEROMAG Medical Equipment Co., Ltd., Hangzhou, China). The device is based on spin-exchange relaxation-free (SERF) technology and incorporates a 64-channel sensor array for whole-head MEG recording. Recordings were performed in a magnetically shielded room with active magnetic field compensation to reduce environmental magnetic interference. The system sensitivity was better than 15 fT/√Hz in the 1–30 Hz frequency range. MEG signals were sampled at 1000 Hz, and the acquisition bandwidth extended to 150 Hz.

Prior to data acquisition, participants were instructed to remove all metallic objects and were positioned in a supine position. The head was stabilized within a customized adult helmet for system calibration. The SERF-MEG system did not include an independent continuous head-motion tracking system. Head movement was minimized by the supine position and helmet stabilization. Recordings with visible movement-related artifacts were excluded during quality control. Head displacement was not quantified continuously. A pattern-reversal visual stimulation paradigm was used. The stimulus was generated using MATLAB R2020a (MathWorks, Natick, MA, USA) and presented through a projection system. The stimulus consisted of a black-and-white checkerboard with equal-area elements and a central fixation point. The viewing distance was 25 cm, and each check measured 0.5 cm, corresponding to approximately 69 arcmin. The checkerboard reversed at a rate of 2.0 reversals/s, and the mean luminance remained constant throughout the experiment. Monocular stimulation was applied, and the non-stimulated eye was occluded with an eye patch. Each eye was recorded in a separate monocular session. Each recording lasted 150 s and included 300 pattern reversals, corresponding to 150 reversal cycles.

VEP recordings were performed using an electrophysiological diagnostic system (RETI-port/Scan 21, Roland Consult, Brandenburg an der Havel, Germany) in accordance with the standards of the International Society for Clinical Electrophysiology of Vision (ISCEV) [6]. Full-field pattern-reversal stimulation was applied with a contrast of 97%. Two check sizes were used (60′ and 15′), with a reversal rate of 1.8–2.2 reversals/s and a viewing distance of 100 cm. P100 latency and N75–P100 peak-to-peak amplitude were analyzed.

MEG and VEP data were collected in separate recording sessions using their respective systems. Both examinations used monocular pattern-reversal stimulation, although the stimulus parameters were specific to each system.

### 2.3. MEG Signal Preprocessing and Feature Extraction

MEG data preprocessing was performed using MATLAB. Raw data were initially band-pass filtered between 1 and 40 Hz to remove low-frequency drifts and high-frequency noise, followed by a 50 Hz notch filter to suppress power-line interference. Independent component analysis (ICA) was used to identify and remove physiological artifacts, including eye movements, blinks, and cardiac activity. Twenty independent components were estimated, and artifact-related components were identified by visual inspection and manually removed. After component rejection, the cleaned signals were reconstructed in the original sensor space. All 64 MEG channels were retained for subsequent analyses.

Continuous MEG recordings were segmented into epochs time-locked to the stimulus triggers, with an analysis window from −100 ms before stimulus onset to 400 ms after stimulus onset. Baseline correction was performed using the −100 to 0 ms pre-stimulus interval. Epochs containing excessive motion artifacts, baseline drifts, or abnormal high-amplitude fluctuations were excluded. Only trials without substantial residual artifacts and with clearly identifiable visually evoked responses were included in subsequent analyses.

Based on the preprocessed data, time-domain features of the VEF were extracted. According to the grand-averaged waveform characteristics, the M100 and M135 components were identified within a 70–200 ms time window. Peak latency was defined as the time point of the maximum local response within the predefined window, and peak amplitude was defined as the amplitude at the corresponding peak relative to the baseline.

To evaluate the overall strength of the multichannel evoked magnetic field, global field power (GFP) was also computed. GFP provides a reference-independent measure of the spatial dispersion of multichannel signals at each time point and does not depend on the selection of a single representative channel [27,28]. The GFP was calculated as follows:

$$\mathrm{GFP}\left(t\right)=\sqrt{\frac{1}{N}\sum_{i=1}^{N}{\left(x_{i}\left(t\right)-\bar{x}\left(t\right)\right)}^{2}}$$

where *N* denotes the number of channels, $\bar{x}\left(t\right)$ represents the instantaneous mean across all channels at time *t*, and $x_{i}\left(t\right)$ denotes the signal of the *i*-th channel at time *t*. Higher GFP values indicate a stronger overall multichannel evoked field response.

**Figure 2:**
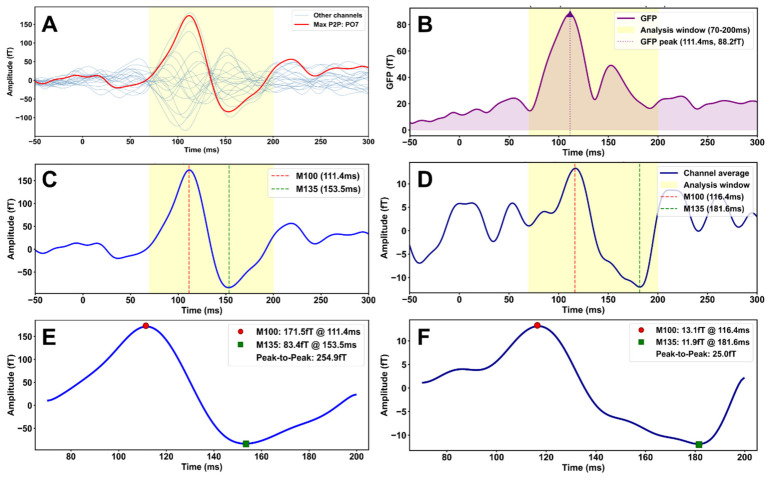
Representative workflow for SERF-MEG feature extraction. Panel (**A**) shows the automatic identification of the channel with the largest peak-to-peak response within the predefined analysis window. Panel (**B**) shows the global field power calculated from the multichannel MEG signals. Panel (**C**) shows the waveform from the selected MaxP2P channel, whereas Panel (**D**) shows the waveform averaged across the valid occipital channels. Panel (**E**) illustrates automatic extraction of M100 latency, M135 latency, and peak-to-peak amplitude from the selected MaxP2P channel. Panel (**F**) illustrates extraction of the corresponding features from the channel-averaged waveform. The shaded regions indicate the predefined analysis window of 70–200 ms. Abbreviations: GFP, global field power; Avg, channel-averaged method; MaxP2P, maximum peak-to-peak channel method.

To reduce the influence of single-channel variability, two complementary feature extraction strategies were adopted: (1) the channel-averaged method (Avg), in which features were averaged across all valid occipital channels to reflect overall occipital cortical responses; and (2) the maximum peak-to-peak channel method (MaxP2P), in which the channel exhibiting the largest peak-to-peak amplitude within the predefined time window was automatically selected as the representative channel to capture the strongest local evoked response. All channel selection procedures were fully automated using custom Python scripts and were applied using identical parameters across all participants (Figure 2). Within the predefined 70–200 ms analysis window, the script automatically identified the channel with the largest peak-to-peak amplitude as the representative MaxP2P channel. The same channel definitions, search window, and peak-detection parameters were applied to all participants.

### 2.4. Paired-Eye and Cross-Modal Statistical Analysis

All statistical analyses were performed in Python 3.10 using the pandas, NumPy, SciPy, and statsmodels packages. Descriptive statistics were obtained for all variables, and continuous data were expressed as mean ± standard deviation (SD). For paired-eye data, differences between the affected and fellow eyes were analyzed using paired t-tests, and effect sizes were computed using Cohen’s d.

To investigate the cross-modal correlations between MEG- and VEP-derived metrics, correlation analyses were performed independently for latency- and amplitude-related measures. Pearson correlation analysis was used to assess linear relationships, while Spearman rank correlation analysis was performed to evaluate nonparametric associations. All correlation analyses were primarily based on interocular difference values (affected eye minus fellow eye) to reduce the influence of inter-individual variability.

In addition, directional concordance analysis was performed. For latency measures, concordance was defined as both VEP and MEG showing prolonged latency in the affected eye compared with the fellow eye. For amplitude measures, concordance was defined as both modalities demonstrating reduced amplitude in the affected eye. The concordance rate was defined as the proportion of participants exhibiting the same direction of change across the two modalities. All statistical tests were two-tailed, and a *p*-value < 0.05 was considered statistically significant.

## 3. Results

### 3.1. Cohort Characteristics

A total of 54 patients with optic nerve injury were included in this study. Among them, 45 patients with unilateral involvement and complete bilateral recordings were eligible for the paired-eye analysis. The remaining nine patients were excluded from the paired analysis due to bilateral involvement (*n* = 2), absence of clear abnormalities in both eyes (*n* = 3), or other reasons (*n* = 4).

In total, electrophysiological recordings from 90 eyes were obtained for both VEP and MEG analyses. The overall distributions of key electrophysiological parameters were as follows: the mean P100 latency of VEP was 113.83 ± 11.24 ms, and the mean P100 amplitude was 8.73 ± 4.65 μV. For MEG-derived metrics, the mean Avg_M100 latency was 105.35 ± 8.51 ms, the mean Avg peak-to-peak amplitude was 108.61 ± 43.72 fT, the mean MaxP2P peak-to-peak amplitude was 215.94 ± 98.16 fT, and the mean GFP was 38.79 ± 16.04 fT.

All participants produced stable and clearly identifiable visually evoked electrophysiological responses, which were deemed suitable for subsequent statistical analyses.

### 3.2. Electrophysiological Alterations in Optic Nerve Injury

Electrophysiological indices were compared between the affected and fellow eyes in the paired-eye cohort (n = 45). Overall, both VEP and MEG showed a consistent pattern of abnormalities in patients with optic nerve injury, characterized by prolonged latencies and reduced amplitudes (Table 1).

**Figure 3:**
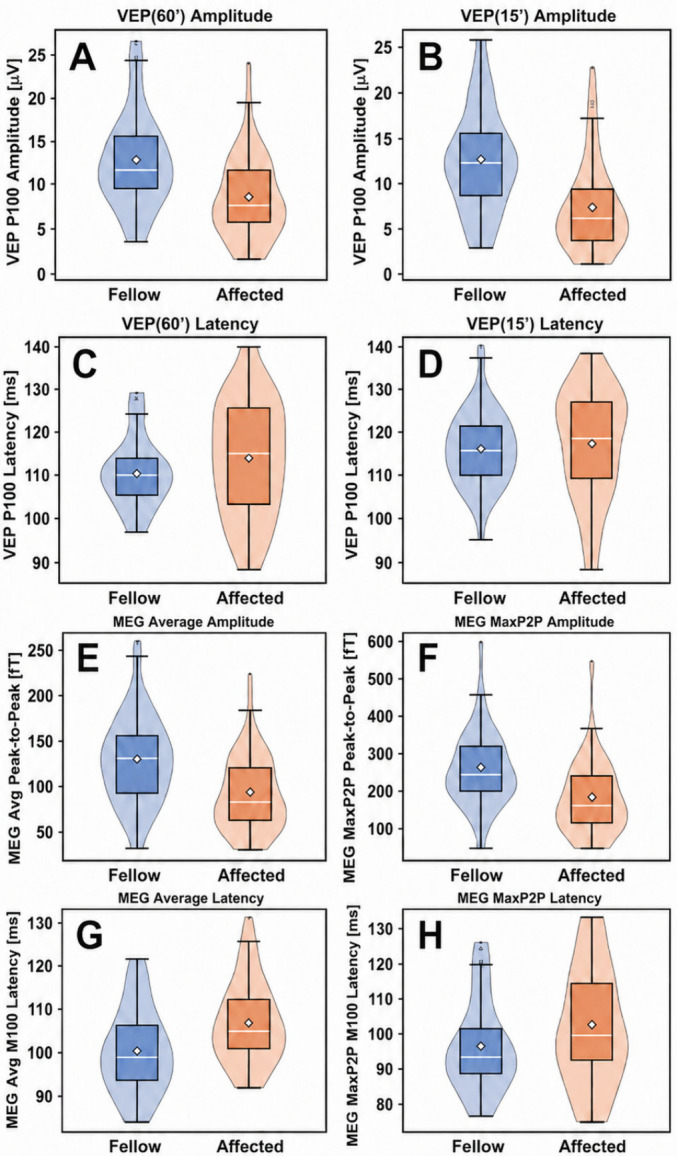
Distribution of VEP and MEG parameters in affected and fellow eyes. Violin plots show the distributions, medians, interquartile ranges, and individual observations of electrophysiological measurements obtained from fellow eyes (Fellow) and affected eyes (Affected). Panels (**A**–**D**) present VEP parameters under 60′ and 15′ checkerboard stimulation, including P100 amplitude and latency. Panels (**E**–**H**) present MEG-derived parameters, including Avg peak-to-peak amplitude, MaxP2P peak-to-peak amplitude, Avg_M100 latency, and MaxP2P_M100 latency. Abbreviations: VEP, visual evoked potential; MEG, magnetoencephalography; Avg, channel-averaged method; MaxP2P, maximum peak-to-peak channel method.

VEP analysis revealed a significant reduction in P100 amplitude in the affected eyes, as shown in Figure 3A–D. Under the large-check (60′) condition, the mean amplitude decreased by 3.09 μV (t = −6.58, *p* < 0.001, Cohen’s d = −0.98). Under the small-check (15′) condition, a similar reduction was observed, with a mean decrease of 4.24 μV (t = −6.19, *p* < 0.001, Cohen’s d = −0.92). In contrast, although P100 latency showed a general trend toward prolongation, no statistically significant differences were observed under either stimulus condition (both *p* > 0.05).

MEG-derived metrics demonstrated a similar pattern of functional impairment. The Avg peak-to-peak amplitude was significantly reduced in the affected eyes (mean difference = −32.97 fT; t = −6.15, *p* < 0.001, Cohen’s d = −0.92). The MaxP2P peak-to-peak amplitude also showed a marked reduction (mean difference = −75.59 fT; t = −5.44, *p* < 0.001, Cohen’s d = −0.81). In addition, global field power (GFP), which reflects the overall strength of the multichannel cortical response, was significantly decreased (mean difference = −9.02 fT; t = −5.63, *p* < 0.001, Cohen’s d = −0.84).

For latency-related MEG measures, the affected eyes exhibited delayed visual responses. The Avg_M100 latency was significantly prolonged (mean difference = 5.18 ms; t = 3.14, *p* = 0.003, Cohen’s d = 0.47), and a similar but less pronounced delay was observed for MaxP2P_M100 latency (mean difference = 5.57 ms; t = 2.08, *p* = 0.043, Cohen’s d = 0.31) (Figure 3E–H).

### 3.3. Cross-Modal Agreement and Correlation Between VEP and MEG

To further investigate the relationship between the two electrophysiological modalities, directional concordance and cross-modal associations between VEP- and MEG-derived indices were systematically analyzed based on paired interocular differences. A total of 45 paired-eye datasets were included, and multiple VEP–MEG metric combinations were analyzed separately under large-check (60′) and small-check (15′) stimulation conditions.

**Figure 4:**
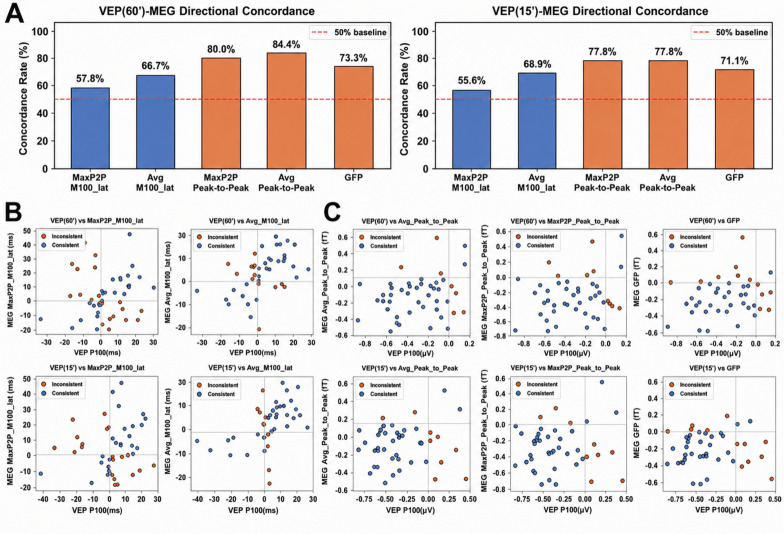
Cross-modal directional concordance analysis between VEP and SERF-MEG measurements. Panel (**A**) summarizes the directional concordance rates between VEP and MEG metrics for latency- and amplitude-related changes under 60′ and 15′ checkerboard stimulation. Panels (**B**,**C**) present scatter plots of paired-eye differences (affected eye − fellow eye) for latency and amplitude measurements, respectively. Blue symbols indicate concordant changes between modalities, whereas orange symbols indicate discordant changes. Abbreviations: GFP, global field power; Avg, channel-averaged method; MaxP2P, maximum peak-to-peak channel method.

Directional concordance analysis demonstrated a moderate level of agreement for latency-related measures (Figure 4A,B). Under the small-check (15′) condition, the highest concordance rate was observed between VEP P100 latency and MEG Avg_M100 latency, reaching 68.9%. Under the large-check (60′) condition, the concordance rate between these two measures was 66.7%. In contrast, the concordance rate between VEP P100 latency and MEG MaxP2P_M100 latency was lower (55.6% and 57.8% under the 60′ and 15′ conditions, respectively).

Overall directional concordance was higher for amplitude-related metrics, with mean concordance rates above 70% (Figure 4A,C). VEP P100 amplitude and MEG Avg peak-to-peak amplitude showed the highest concordance rates, at 84.4% under the large-check (60′) condition and 77.8% under the small-check (15′) condition. Under the 60′ and 15′ conditions, the concordance rates between VEP P100 amplitude and MEG MaxP2P peak-to-peak amplitude were 80.0% and 77.8%, respectively, while the corresponding rates for GFP were 73.3% and 71.1%. Overall, these results indicate that VEP and MEG showed greater directional concordance for amplitude-related changes than for latency-related changes.

Correlation analysis also revealed significant positive correlations between the interocular differences in latency-related measures (Table 2). The Pearson correlation coefficient between the VEP P100 latency difference and the MEG Avg_M100 latency difference under the large-check (60′) condition was 0.41 (*p* = 0.005), and the corresponding Spearman rank correlation coefficient was 0.45 (*p* = 0.002). Similar significant associations were found under the small-check (15′) condition (Pearson r = 0.38, *p* = 0.010; Spearman ρ = 0.44, *p* = 0.003). In contrast, the VEP P100 latency difference was not significantly correlated with the MEG MaxP2P_M100 latency difference (all *p* > 0.05).

VEP- and MEG-derived indices did not show statistically significant correlations for amplitude-related parameters in either the Pearson or Spearman correlation analyses (all *p* > 0.05). These results suggest that amplitude-related alterations did not show a consistent quantitative linear relationship across the two modalities, despite high directional concordance.

### 3.4. Comparison of MEG Feature Extraction Strategies

**Figure 5:**
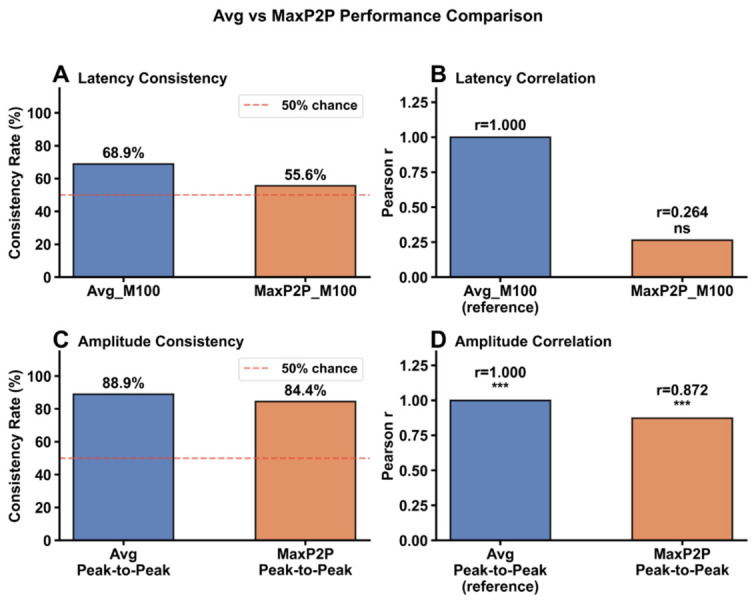
Comparison of MEG feature extraction strategies. Panels (**A**,**C**) compare the directional concordance rates between VEP-derived and MEG-derived latency- and amplitude-related measures obtained using the channel-averaged (Avg) and maximum peak-to-peak channel (MaxP2P) methods. Panels (**B**,**D**) illustrate the relationships between corresponding latency and amplitude metrics extracted using the two methods. Abbreviations: Avg, channel-averaged method; MaxP2P, maximum peak-to-peak channel method. *** *p* < 0.001.

The Avg and MaxP2P, two MEG feature extraction techniques, were further compared, and the Avg approach showed more consistent cross-modal performance overall. In latency analysis, both agreement and correlation between Avg_M100 and VEP P100 latency were higher than those observed for MaxP2P_M100. In amplitude analysis, the Avg-based peak-to-peak measures also showed higher directional concordance with VEP-derived amplitudes. In contrast, although the MaxP2P approach captured the strongest local responses, it exhibited greater inter-individual variability (Figure 5).

Overall, these results demonstrate that VEP and MEG show significant correlations and moderate cross-modal concordance in detecting visual conduction delay associated with optic nerve injury. For amplitude-related measurements, however, the two modalities mainly show directional concordance rather than strong quantitative correlations.

## 4. Discussion

In this study, we comprehensively analyzed visually evoked responses in patients with optic nerve injury using comparable SERF-MEG and conventional VEP paradigms, with a focus on cross-modal concordance in detecting visual pathway dysfunction. The results demonstrated a consistent pattern of abnormalities in both modalities, characterized by reduced response amplitudes and prolonged latencies. For instance, MEG-derived M100 latency exhibited a consistent association with VEP P100 latency, whereas amplitude-related measures largely indicated directional concordance rather than strong quantitative correlations. Furthermore, compared with the MaxP2P method based on single-channel peak responses, the channel-averaged method displayed greater stability in cross-modal comparisons. These findings suggest that SERF-MEG may be used in conjunction with conventional VEP for neuro-ophthalmic functional assessment and may reliably detect visual pathway abnormalities associated with optic nerve injury.

The moderate cross-modal concordance between VEP and MEG in latency-related measures is one of the study’s main findings. Specifically, there was a significant correlation between Avg_M100 latency and VEP P100 latency, and similar findings were observed under both stimulus conditions. Latency measures are generally considered relatively stable and physiologically meaningful indicators of visual pathway function, primarily reflecting visual conduction time along the visual pathway from the optic nerve through the optic radiation to the occipital visual cortex. This conduction process is closely related to myelin integrity and axonal conduction efficiency. In ON, ION, and TON, demyelination, axonal edema, ischemic injury, and secondary neurodegeneration can all contribute to delayed visual conduction. Therefore, latency prolongation represents a well-established electrophysiological hallmark of visual pathway dysfunction.

In contrast, although amplitude-related measures demonstrated relatively high directional concordance between the affected and fellow eyes, their quantitative correlations across modalities were limited. This discrepancy may be related to the physiological and technical properties of amplitude-based measures. Unlike latency, which mainly reflects visual conduction time, response amplitude depends on the strength and temporal synchronization of the underlying neuronal activity. It can also be affected by attentional state, fixation, signal-to-noise ratio, and individual anatomical differences [6,29]. In addition, VEP amplitude may be influenced by electrode placement and impedance, whereas MEG amplitude may vary with head position and the distance between the sensors and the cortical sources [6,30].

In addition, VEP recordings are derived from scalp-recorded electrical potentials, whereas MEG records magnetic fields generated by intracellular neuronal currents. These two modalities differ in their biophysical generation mechanisms and in their sensitivity to the spatial orientation of neuronal sources [31,32]. Therefore, even when concordant directional changes are observed across modalities under pathological conditions, a strict linear correspondence between amplitude measures may not be expected.

Previous studies have demonstrated that MEG provides millisecond temporal resolution comparable to that of conventional VEP, while offering improved spatial localization and multichannel coverage of cortical activity. These features enable a more comprehensive characterization of cortical neural dynamics. Unlike VEP, which is limited by the spatial constraints of scalp electrode configurations, MEG can more effectively capture the spatiotemporal patterns of synchronized activity in the visual cortex and has therefore been widely applied in studies of sensory processing, cortical network function, and functional reorganization [33,34].

Although the present study primarily focused on time-domain evoked response metrics derived from SERF-MEG and did not include source localization or functional connectivity analyses, the significant reduction in GFP observed in affected eyes suggests that SERF-MEG may provide information beyond conventional VEP measures by capturing alterations in the overall strength of multichannel cortical responses. These findings further support the potential value of SERF-MEG as a complementary functional assessment tool in neuro-ophthalmology.

In the evaluation of different MEG feature extraction methods, the present study demonstrated that the channel-averaged method showed more stable cross-modal performance than the MaxP2P method. Specifically, the Avg-derived features displayed improved performance in terms of latency correlation, amplitude concordance, and overall stability. In comparison, although the MaxP2P method was able to capture the channel with the largest local response, it was more susceptible to local noise, inter-individual variability, and occasional peak fluctuations. These findings suggest that, in cross-modal comparisons, features derived from channel-averaged cortical responses may provide greater robustness than single-channel extremum-based measures, making them more suitable for group-level concordance assessments. This observation has methodological implications for the standardization of feature extraction methods in SERF-MEG studies.

An additional strength of this study is the use of a paired-eye analytical design. Given that both MEG and VEP amplitude measures are influenced by inter-individual factors such as head shape, cortical anatomy, volume conduction properties, and baseline neural activity, direct inter-subject comparisons are often associated with substantial variability. By using within-subject paired-eye difference analysis, the influence of inter-subject heterogeneity was substantially reduced, thereby improving the statistical sensitivity and stability of cross-modal comparisons. In the context of optic nerve injury, this design allows a more direct assessment of disease-related interocular functional asymmetry and facilitates the evaluation of directional concordance between different electrophysiological modalities in detecting visual pathway dysfunction.

Patients with TON, ION, and ON were included in this study. Although the underlying pathological mechanisms of these conditions vary (inflammatory demyelination in ON, ischemic axonal injury in ION, and mechanical injury with secondary neurodegeneration in TON), they all ultimately lead to reduced cortical visual responses and impaired visual conduction. Therefore, the present study focused on shared patterns of visual pathway dysfunction across etiologies rather than disease-specific electrophysiological differences. This design supports the evaluation of SERF-MEG as a broadly applicable functional assessment tool across heterogeneous optic nerve disorders.

Several limitations should be acknowledged. First, the relatively small sample size and single-center design, together with imbalanced distributions across disease subgroups, limited further stratified analyses by etiology. Second, the present study primarily focused on time-domain evoked response metrics and did not incorporate source localization, frequency-domain features, functional connectivity, or graph-theoretical analyses. As a result, the current understanding of cortical functional reorganization in optic nerve injury remains limited. Third, the SERF-MEG system did not provide continuous head-motion tracking. Although head movement was minimized by the supine position and helmet stabilization, residual movement could not be quantified and may have influenced signal quality. In addition, this study mainly examined cross-modal concordance between MEG and VEP, without establishing longitudinal relationships with clinical visual function measures, disease progression, or long-term prognosis. Finally, because the present study did not directly compare SERF-MEG with conventional SQUID-MEG or perform a formal cost analysis, the reported sensor sensitivity should not be interpreted as evidence of technical or economic superiority; future comparative studies are required to assess the relative performance and cost-effectiveness of the two technologies.

Future studies integrating optical coherence tomography, visual acuity, functional visual assessments, and multimodal brain network analyses are warranted to construct a more comprehensive framework for visual pathway assessment. Moreover, SERF-MEG-based functional connectivity analysis, brain network reorganization modeling, and machine learning-assisted classification may further improve the differential diagnosis of optic nerve disorders. With ongoing improvements in system miniaturization and clinical accessibility, SERF-MEG may also hold promise for screening, treatment–response monitoring, and longitudinal follow-up in neuro-ophthalmic disorders.

## 5. Conclusions

This study showed that SERF-MEG detected reduced cortical response amplitudes and prolonged M100 latency in eyes affected by optic nerve injury. Avg M100 latency showed moderate directional concordance and significant correlations with VEP P100 latency, whereas amplitude measures showed similar directions of change but limited quantitative correlations across modalities. The channel-averaged method showed more stable cross-modal performance than the MaxP2P method. These findings provide preliminary validation of SERF-MEG for detecting visual pathway dysfunction associated with optic nerve injury. Its consistent abnormalities and significant latency correlations with conventional VEP support its potential use as an objective functional assessment tool, while its multichannel cortical measures may provide information beyond that obtained from conventional VEP. Further studies with larger and more diverse cohorts are needed to determine its clinical utility in visual pathway disorders.

## Figures and Tables

**Table 1:** Paired comparison between affected and fellow eyes.

Variable	Fellow Eye	Affected Eye	Mean Difference	*p* Value	Cohen’s d
P100 (60′) (ms)	111.49 ± 7.85	114.17 ± 13.03	2.68 ± 13.05	0.175	0.21
P100 (60′) (μV)	10.27 ± 4.46	7.18 ± 4.22	−3.09 ± 3.15	<0.001 ***	0.98
P100 (15′) (ms)	117.71 ± 8.46	118.85 ± 11.95	1.14 ± 14.31	0.596	0.08
P100 (15′) (μV)	11.54 ± 5.47	7.30 ± 5.01	−4.24 ± 4.59	<0.001 ***	0.92
Avg Peak-to-Peak (fT)	125.09 ± 45.08	92.12 ± 40.36	−32.97 ± 35.99	<0.001 ***	0.92
MaxP2P Peak-to-Peak (fT)	253.73 ± 100.04	178.14 ± 93.28	−75.59 ± 93.18	<0.001 ***	0.81
GFP (fT)	43.30 ± 15.33	34.28 ± 15.75	−9.02 ± 10.74	<0.001 ***	0.84
Avg M100 (ms)	102.75 ± 8.92	107.94 ± 8.00	5.18 ± 11.07	0.003 **	0.47
MaxP2P M100 (ms)	96.88 ± 13.61	102.45 ± 18.04	5.57 ± 17.93	0.043 *	0.31

* *p* < 0.05, ** *p* < 0.01, and *** *p* < 0.001. Mean differences were calculated as affected eye minus fellow eye.

**Table 2:** Cross-modal directional concordance and correlation analysis.

VEP Variable	MEG Variable	Concordance (%)	Pearson r	*p*	Spearman ρ	*p*
P100 latency (60′)	Avg_M100_lat	66.7	0.41	<0.01 **	0.45	<0.01 **
P100 latency (15′)	Avg_M100_lat	68.9	0.38	0.01 *	0.44	<0.01 **
P100 latency (60′)	MaxP2P_M100_lat	57.8	0.14	0.34	0.14	0.36
P100 latency (15′)	MaxP2P_M100_lat	55.6	0.13	0.40	0.14	0.35
P100 amplitude (60′)	Avg_Peak-to-Peak	84.4	0.25	0.10	0.21	0.17
P100 amplitude (15′)	Avg_Peak-to-Peak	77.8	0.05	0.73	0.05	0.72
P100 amplitude (60′)	MaxP2P_Peak-to-Peak	80.0	0.20	0.19	0.11	0.47
P100 amplitude (15′)	MaxP2P_Peak-to-Peak	77.8	0.06	0.71	0.04	0.80
P100 amplitude (60′)	GFP	73.3	0.23	0.13	0.19	0.22
P100 amplitude (15′)	GFP	71.1	-0.02	0.89	0.06	0.68

* *p* < 0.05, and ** *p* < 0.01. Correlations were calculated using interocular difference values defined as affected eye minus fellow eye. Directional concordance was defined as the proportion of patients showing the same direction of change in VEP and MEG measures.

## Data Availability

The data presented in this study are available on request from the corresponding author. The data are not publicly available due to patient privacy and ethical restrictions.

## Abbreviations

The following abbreviations are used in this manuscript:

SERF-MEG	Spin-exchange relaxation-free magnetoencephalography
VEPs	Visual evoked potentials
ON	Optic neuritis
ION	Ischemic optic neuropathy
TON	Traumatic optic neuropathy
MEG	Magnetoencephalography
SERF	Spin-exchange relaxation-free
VEFs	Visual evoked fields
SQUIDs	Superconducting quantum interference devices
ISCEV	International Society for Clinical Electrophysiology of Vision
ICA	Independent component analysis
GFP	Global field power
Avg	Channel-averaged method
MaxP2P	Maximum peak-to-peak channel method
SD	Standard deviation
